# Toxins of Prokaryotic Toxin-Antitoxin Systems with Sequence-Specific Endoribonuclease Activity

**DOI:** 10.3390/toxins9040140

**Published:** 2017-04-14

**Authors:** Hisako Masuda, Masayori Inouye

**Affiliations:** 1 School of Sciences, Indiana University Kokomo, Kokomo, IN 46902, USA; 2 Department of Biochemistry, Robert Wood Johnson Medical School, Rutgers University, New Brunswick, NJ 08854, USA

**Keywords:** toxin-antitoxin systems, sequence-specific endoribonucleases, bacterial stress response, growth arrest

## Abstract

Protein translation is the most common target of toxin-antitoxin system (TA) toxins. Sequence-specific endoribonucleases digest RNA in a sequence-specific manner, thereby blocking translation. While past studies mainly focused on the digestion of mRNA, recent analysis revealed that toxins can also digest tRNA, rRNA and tmRNA. Purified toxins can digest single-stranded portions of RNA containing recognition sequences in the absence of ribosome in vitro. However, increasing evidence suggests that in vivo digestion may occur in association with ribosomes. Despite the prevalence of recognition sequences in many mRNA, preferential digestion seems to occur at specific positions within mRNA and also in certain reading frames. In this review, a variety of tools utilized to study the nuclease activities of toxins over the past 15 years will be reviewed. A recent adaptation of an RNA-seq-based technique to analyze entire sets of cellular RNA will be introduced with an emphasis on its strength in identifying novel targets and redefining recognition sequences. The differences in biochemical properties and postulated physiological roles will also be discussed.

## 1. Introduction

**Figure 1:**
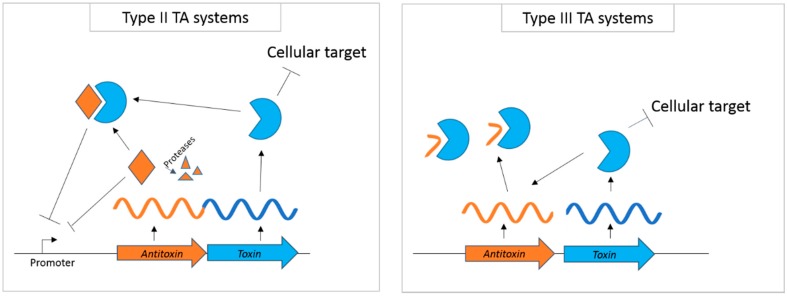
Type II and III toxin-antitoxin (TA) systems. Toxins and antitoxin genes are represented by blue and orange thick arrows. Wavy lines represent mRNA that are transcribed from each module. In Type II TA systems, toxins and antitoxins are co-transcribed. Antitoxins form complexes with toxin proteins under preferred growth conditions. Antitoxins are degraded by stress-inducible proteases, such as Lon. Antitoxin and the TA complex can negatively regulate expression by binding to their own promoter region. In Type III TA systems, antitoxin RNA is processed by toxins that have nuclease activity. Both processed and unprocessed antitoxins remain bound to the toxin’s active site, inhibiting toxins from digesting other cellular RNAs.

Toxin-antitoxin (TA) systems are ubiquitous modules found in almost all sequenced bacterial genomes [1,2]. Toxins are small polypeptides that negatively regulate cellular growth by inhibiting essential cellular processes, such as cell division, DNA replication, protein translation and membrane integrity [3,4,5,6,7]. Many genomes carry multiple copies of TA pairs, often each differing in the mechanisms of growth inhibition and expression patterns [1,2,8]. The transcription of a toxin gene is often coupled with its cognate antitoxin with these two genes typically forming an operon. Toxin activity is blocked by antitoxins when approximately an equivalent amount of toxin and antitoxin is present in a cell. Lowering the antitoxin concentration allows for the increase in free toxins, which leads to the arrest of cell growth. TA systems are classified into six subgroups based on the type of antitoxin (e.g., protein or RNA) and the mechanism of how antitoxin neutralizes toxin activity [9]. For example, in Type II TA systems, antitoxin proteins sequester toxins via physical interactions (Figure 1). Preferential digestion of antitoxin proteins by stress-inducible proteases frees toxins to exert their toxicity. In Type III TA systems, sRNA antitoxin is both a substrate and the inhibitor of the cognate endoribonuclease toxin. In type V TA systems, the antitoxin is a nuclease that specifically digests toxin mRNA to repress expression [10].

The vast majority of known TA toxins compromise protein translation [1,11]. TA toxins inhibit translation through multiple mechanisms, including sequence-specific digestion of RNAs (rRNA, mRNA, tRNA, sRNA and/or tmRNA), ribosome-dependent cleavage of mRNA, non-specific digestion of mRNA and the inhibition of ribosome assembly [1,12,13,14]. Ribosome-dependent endoribonucleases, such as RelE, YoeB and YafO, do not cleave mRNA in the absence of ribosomes, as toxins themselves have zero or little nuclease activity [13,15,16,17]. Upon association with a ribosome, toxins induce mRNA cleavage within the ribosomal A site immediately downstream of the start codon, or at 11 to 13 bases downstream of the initiation codons [4,13,17,18]. Toxin YafQ of DinJ-YafQ is capable of mRNA cleavage in the absence of ribosome; however, sequence- and frame-specific cleavage occurs only in the presence of ribosomes [19]. In contrast, ribosome independent nucleases digest RNA in a sequence-specific manner even in the absence of ribosomes. While studies of ribosome-dependent inhibitions of translation and non-specific digestion of mRNA also have scientific merit, this review will focus specifically on the discovery and studies of toxins with ribosome-independent, sequence-specific ribonuclease activity.

MazF from *Escherichia coli* (MazF-ec) is the first TA toxin to have been identified as a sequence-specific ribonuclease [3,20]. The original methods employed to characterize MazF-ec have been adapted to characterize a myriad of TA toxins with similar activities [3,21,22]. The initial discovery of MazF-ec inhibiting protein synthesis was achieved by monitoring the incorporation of ^35^S-labeled methionine into newly-synthesized peptides [3,22]. Upon ectopic expression of MazF-ec, the synthesis of proteins halts immediately [3]. In contrast, the rates of DNA replication and RNA synthesis do not change for a prolonged period, indicating that the direct target of MazF-ec is protein synthesis. Furthermore, Northern blot analysis confirmed the immediate disappearance of cellular mRNA following MazF-ec expression [3]. MALDI-mass spectrometry analysis of the product of MazF-ec-digested mRNA confirmed that MazF-ec is an endoribonuclease that specifically cleaves at the phosphodiester linkage [23].

The specific cleavage sites of MazF-ec and other endoribonucleases have been characterized by the in vivo expression of toxins followed by the primer extension of extracted RNA and/or by in vitro digestion of synthesized RNA by purified toxin protein [3,20]. In all tested cases, sequence-specific ribonucleases can only cleave single-stranded RNA and do not cleave RNA/RNA or RNA/DNA duplexes [3,21]. The accessibility of the recognition sequence to the enzyme also seems crucial, as the sequences that are located within a secondary structure or buried within a large protein-RNA complex (e.g., ribosome) are not susceptible to cleavage [24,25,26]. The length of the cleavage sequence of MazF homologues varies from three to seven bases [1,3,27,28]. For example, MazF-ec recognizes a triplet, ACA [3]. MazF homologues from Gram-positive *Staphylococcus aureus* and *Bacillus subtilis* cleave UACAU [27,29,30,31]. MazF-hw from archaea *Haloquadratum walsbyi* recognizes a seven-base sequence (UUACUCA) [28]. Relatively long RNA (e.g., MS2 RNA) has often been used as a substrate to analyze the sequence specificity of ribonucleases that recognize bases longer than five. However, because of the limited number of pentamers represented in one type of RNA substrate, identifying the exact consensus sequence could not be achieved in some cases [21,24]. Recently, the RNA-seq-based method of detecting cleavage sites in all cellular RNAs (MORE RNA-seq) has been adopted. Due to the extensive sequencing depth, the technique allows for the refinement of the recognition sequences, as well as for the detection of novel RNA targets, such as tRNA, rRNA and tmRNA [24,26].

The position and the frame of cleavage sequences within the mRNA seem to provide another layer of specificity for digestion in vivo. Vesper and colleagues demonstrated that MazF-ec cleaves ACA at or near the AUG start codon, producing leaderless mRNA (lmRNA) [32]. Furthermore, MazF-ec digests ACA sites only in the reading frame, while ACA sites that occurred out of the frame were not cleaved [33]. Coincidently, ribosome-dependent ribonucleases YoeB and RelE also showed a similar reading-frame-dependent cleavage preference [13,17,34]. *E. coli* RelE preferentially digests a stop codon (UAG) within the ribosomal A site [13]. *E. coli* and *S. aureus* YoeB cleaves mRNA at three bases downstream of the initiation codon without sequence specificity [17,34]. The digestion of MazF with reading-frame dependency suggests that although these enzymes can cleave RNA in a ribosome independent manner, the majority of in vivo digestion would occur in association with the ribosome. Preferential digestion near translational initiation sites also suggests the differential importance of translation initiation, elongation and termination processes on ribonuclease digestion. Oron-Gottesman and colleagues postulated that the binding of MazF-ec to an extracellular-death-factor (EDF)-like element in the ribosomal protein bS1 is important for the RNA cleavage activity of MazF [33]. Further research is needed to decipher whether all of the sequence-specific ribonucleases cleave predominantly at the 5′ and/or 3′ end of mRNA and if it is indeed associated with a ribosome in vivo.

While earlier in vivo digestion studies of sequence-specific ribonucleases were mainly focused on the digestion of mRNA, recent studies have shown that some toxins can also digest other types of RNA, such as rRNA, tRNA, sRNA and tmRNA [2,4,24,25,26,32,35]. MazF-ec cleaves 16S rRNA, producing stress ribosomes that specifically translate leader-less mRNAs [32]. A homologue of MazF in *Mycobacterium tuberculosis*, MazF-mt3, cleaves the helix/loop 70 of 23S rRNA and the anti-Shine-Dalgarno (aSD) sequence of 16S rRNA, which also appears to allow a selective translation of leaderless mRNA in *Mycobacterium* [26]. The digestion of 23S rRNA at the single-stranded region of helix/loop 70 is also catalyzed by another MazF homologue in *M. tuberculosis*, MazF-mt6 [24]. A phylogenetically unrelated ribonuclease, VapC, can digest 23S rRNA, but it cleaves a separate region of the 23S rRNA, the sarcin-ricin loop [36]. In addition, VapC from *Shigella flexneri* 2a and MazF-mt9 of *M. tuberculosis* digest tRNA in a sequence- and likely in a structure-dependent manner [25,35].

It is interesting to note that unlike the digestion of mRNA, the digestion of tRNA and rRNA occurs primarily at one or two sites [2,24,25,26,32,35]. In *E. coli*, two major stable digestion products of 16S rRNA were detected following incubation with MazF [37]. Furthermore, the authors have shown that these digested 16S rRNA products are stable in vivo and can be reverted back to a full size, functional unit by RNA ligase (RtcB) [37]. The reversibility of rRNA cleavage must be included in future studies of the toxin’s control of translation.

In Type II TA systems, antitoxin proteins bind their own promoter region and inhibit transcription [38,39]. Toxin and antitoxin proteins form a complex, which can also function as a transcriptional repressor. In many TA systems, the strength of binding between the DNA and TA complex differs based on the stoichiometric ratio of toxins and antitoxins, exhibiting conditional cooperativity [40]. Transcription is strongly inhibited at intermediate toxin/antitoxin ratios, while transcription is allowed at both high and low ratios. As some TA systems can regulate the transcription of genes other than their own, altering concentrations of toxin and/or antitoxin seems to have an effect on global gene expression patterns [41,42].

The scope of this review is to summarize the biochemical properties of known TA toxins with sequence-specific ribonuclease activities. It should be noted that toxins with such activities thus far are classified as Type II or III TA systems (Table 1), but not all Type II and III toxins are nucleases. Various homologues of ribonucleases will be introduced with a focus on sequence specificity and the types of RNA substrates. Furthermore, their roles in bacterial physiology, either caused directly by the digestion of RNA or by altering transcriptional regulatory activities of TA complexes, will be discussed.

## 2. Families of Sequence-Specific Endoribonuclease TA Toxins

Protein sequence and structure comparisons revealed some homology among TA ribonuclease toxins (Table 1). Many toxins, including ChpBK, Kid and ToxN, are homologous to MazF, while others belong to distinctive families [1]. For example, VapC is a homologue of T4 RNase H with a PIN domain, while MqsR and YhaV belong to the RelE superfamily. With the exception of ToxN and AbiQ (Type III TA systems), other ribonucleases are toxins of Type II TA systems, sharing a similar mode of transcriptional regulation.

TA toxins with endoribonuclease activity are found in a wide range of prokaryotic taxa, ranging from Gram-negative and positive-bacteria to archaea. Many prokaryotic genomes contain multiple copies of sequence specific ribonucleases [1,2,8]. For example, the *E. coli* K-12 genome carries MazF, ChpBK, MqsR, YhaV, HicA and RnlA (Table 1). The pathogen *M. tuberculosis* contains nine copies of MazF homologues (e.g., MazF-mt1, -mt3, -mt6, -mt7 and -mt9), each with a different sequence specificity [8,21,24,26,45,50]. Here, we summarize the biochemical properties of the nucleases from *E. coli*, as well as other prokaryotic organisms [21,22,24,26,27,30,31,45,50,51].

### 2.1. MazF-ec

MazF-ec is the toxin of the best studied Type II TA system, *mazEF* on the *E. coli* chromosome [3,20,23]. MazF-ec recognizes the single-stranded RNA triplet sequence (ACA) and cleaves after the first A-residue (denoted as A^CA with ^ indicating the cleavage site) [3]. Pulse labeling experiments have shown that the production of a majority of the cellular proteins ceases when MazF-ec is expressed in vivo [52]. As almost all mRNA contain ACA triplets, the authors postulated that MazF-ec cleaves nearly all of the cellular mRNA in a ribosome-independent manner, thereby halting cellular protein synthesis. The continued production of a protein whose transcript is engineered to be devoid of ACA suggests that the protein synthetic process itself remains operational following MazF overproduction [52,53].

While MazF-ec cleaves all available ACA sites in vitro, a recent RNA-seq analysis of the cellular RNA following MazF-ec overproduction identified a preferential digestion of ACA sites at or closely upstream of the AUG start codon to produce short-leaderless mRNA (lmRNAs) [32].

In addition to mRNA, the digestion of 16S rRNA by MazF was detected in recent studies [32,54]. The ACA site at the 5′ of helix 45 is prone to degradation by MazF-ec. Upon cleavage, 43 nucleotides from the 3′ terminus, including the anti-Shine-Dalgarno (aSD) sequence, are removed from the 16S rRNA. The ribosome devoid of aSD is called the stress ribosome (70S^Δ43^). Due to the lack of aSD, the stress ribosome cannot initiate translation of full-length mRNA [32]. However, the stress ribosomes remain assembled and retain their ability to translate mRNA that lacked SD (lmRNA). Originally, the stress ribosomes were postulated to translate specific sets of transcripts. However, later studies with *Mycobacteria* identified an association of a variety of transcripts with the stress ribosomes [32,54].

Mets et al. have recently claimed that MazF and MqsR induce rRNA cleavages at multiple sites in vivo [55]. RNA-seq analysis identified that some cleavage had occurred at sequences that were not recognized by MazF and MqsR, suggesting an induction of one or more additional nucleases. These results also imply that MazF does not digest 16S rRNA within the fully-assembled 70S ribosome, but cleaves excess rRNA precursors.

The extent of 16S rRNA digestion in vivo, as well as its physiological significance remain enigmatic. Earlier studies reported a lack of 16S rRNA digestion by MazF-ec, while later studies detected extensive digestion as discussed above [3,32,54]. Suzuki et al. demonstrated the ability of MazF-expressing cells to synthesize a desired peptide from ACA-less transcripts [32,52,53]. Successful translations of ACA-less transcripts in MazF-producing cells seem impossible if cells are completely devoid of fully-assembled ribosomes with aSD [52,53]. Fractions of complete 70S ribosomes present in MazF-expressing cells must be carefully examined in future studies.

Structural studies of the MazEF complex revealed that one MazE dimer binds two MazF dimers, forming a 2:4 hetero-hexameric complex [38]. The interaction between MazE and MazF is mediated by the unstructured C-term extension of MazE, which binds to the dimer of MazF. The highly negative charge of the MazE C-term extension is thought to mimic RNA and binds the active site of MazF [3,23]. Therefore, upon the binding of MazE, the catalytic activity of MazF is inhibited. There is a negative cooperativity between two RNA binding sites in the MazF homodimer [56]. The binding of one MazE to one of the two RNA binding sites within a MazF dimer efficiently inhibits the catalytic activity of both sites.

Like other Type II TA systems, MazE by itself, as well as the MazEF complex function as transcriptional repressors of their own expression [57]. The 5′ UTR of the *mazEF* loci contains two promoters with alternating palindromes. MazE represses the transcription more weakly than the MazEF complex, suggesting that the change in MazEF stoichiometry affects its expression patterns. The antitoxin MazE is preferentially degraded by stress-inducible proteases Lon and ClpP. Thereby, transcription of the *mazEF* operon is induced by various stress conditions [15,58].

### 2.2. MazF Homologues from M. tuberculosis

*M. tuberculosis* contains nine copies of MazF, each with a distinctive sequence specificity (Table 1). The primer extension of *era* mRNA in vivo determined that MazF-mt1 cleaves between the U and A-residue of CU^ACC [21]. The result was confirmed by in vitro digestion using purified MazF-mt1. Another minor cleavage sequence (UU^ACA) was also identified by in vitro assay. The in vitro digestion of shorter synthetic RNA revealed a flexible substrate range of MazF-mt1. The study with 15-base RNA demonstrated that the consensus sequence is U^AC, while a study using an 11-base RNA showed that any single base in UAC can be changed to another base and still be digested [21]. Similar flexibility was also observed for MazF-mt7. Most cleavage occurs with U^CGCU with one or two base alterations [45]. Although all cleavable sequences include a third G-residue, less stringent specificity has been observed for the other positions.

Recent RNA-seq-based screening of the entire cellular mRNA revealed that the cleavage sequence for MazF-mt3 is also five bases long, and the recognition sequence is U^CCUU [26]. MazF-mt3 is also found to digest 23S and 16S rRNA [26]. Digestion of 23S rRNA occurs at the U^UCCU sequence within the single-stranded area of the helix/loop 70. This region of 23S rRNA serves multiple important functional roles of the ribosome. The region is crucial for ribosome assembly, as a single mutation in this sequence abolishes the association between 50S and 30S ribosomal subunits [59]. The region is also essential for the interaction with tRNA and with the ribosome recycling factor RRF [60]. As the recognition sequence is localized in the region that is crucial for ribosome assembly, the digestion by MazF-mt3 must lower the number of fully-functional ribosomes in the cell. Additionally, MazF-mt3 also digests 16S rRNA. Cleavage of 16S rRNA occurs at the anti-Shine-Dalgarno sequence [26]. Such loss is postulated to cause the selective translation of leaderless mRNA in *M. tuberculosis* in a similar manner as 16S rRNA digestion by MazF-ec in *E. coli.* Although significant digestion has been detected with both in vivo and in vitro experiments, the digestion efficiency in vivo seems to be weak, as a majority of rRNA remains intact after 1 h of MazF-mt3 expression [26].

While the original biochemical study of MazF-mt6 using *era* mRNA appeared to show that this enzyme has a non-stringent sequence specificity, a recent RNA-seq-based study revealed that the consensus sequence is UU^CCU [21,24]. MazF-mt6 also has activity towards 23S rRNA [24]. It digests the recognition sequence in helix/loop 70 of 23S rRNA [24]. This UUCCU signature is conserved in ribosomes from many taxa, suggesting its functional importance. In vitro protein synthesis of mRNA lacking a UUCCU motif is still blocked by the addition of MazF-mt6, indicating that MazF-mt6 inhibits translation not only via digestion of mRNA, but also due to rRNA digestion [24]. The ectopic expression of MazF-mt6 reduces the amount of full-length 23S rRNA, demonstrating that the digestion of rRNA also occurs in vivo. The authors further showed that MazF-mt6 cleaves 23S rRNA in the 50S ribosome, but does not digest 23S rRNA in the 70S ribosome [24]. Their results suggest that MazF-mt6 inhibits the assembly of 70S ribosomes, while it has no effect on pre-existing 70S complexes.

With the RNA-seq analysis, MazF-mt9 is shown to digest two specific types of tRNA, as well as mRNA at UU^U [25]. The majority of the tRNA in *M. tuberculosis,* totaling 45, are resistant to cleavage by MazF-mt9, despite the presence of a recognition sequence. Digestion is limited to the UUU within the single stranded regions of tRNA. Only two types of tRNA are digested by MazF-mt9; the D-loop of tRNS^Pr^°^14^ and the single-stranded region within the tRNA^Lys43^ anticodon [25].

### 2.3. MazF Homologues from Other Gram Positive Bacteria

Various other members of Gram-positive bacteria carry MazF homologues [27,29,30,31]. MazF-bs from *B. subtilis* and MoxT from *B. anthracis* share a 94% sequence identity. Both enzymes recognize and cleave the same five-base sequence U^ACAU [27,31]. MazF from pathogenic and non-pathogenic *Staphylococcus* strains, *S. aureus* and *S. equorum*, also exhibits identical sequence specificity (U^ACAU) [29,30].

### 2.4. MazF-mx

*M. xanthus* is a Gram-negative bacterium that forms a multicellular structure called a fruiting body. MazF-mx was originally shown to be responsible for altruistic cell death during the formation of the fruiting body; however, its direct involvement is currently under debate [46,61,62]. While many antitoxins of TA systems form an operon with their cognate toxins, the antitoxin gene of MazF-mx is distantly located on the genome. The antitoxin (MrpC) was identified through a yeast two-hybrid screening, and its antitoxin activity was subsequently verified [46].

MazF-mx cleaves GU^UGC [46]. The first G-residue can be replaced by an A-residue. Based on the primer extension using MS2 RNA as a template, the enzyme has a preference for GAGU^UGCA over other sequences containing GUUGC. Higher concentrations of enzymes allow for the detection of additional cleavage sites, such as AUGU^CAGG, ACGU^AAUA and ACGU^AAAG.

### 2.5. MazF-hw

MazF-hw is found in the halophilic archaea, *Haloquadratum walsbyi* [28]. It was the first endoribonuclease discovered that recognized a seven-base sequence (UU^ACUCA). UUACUCA is one of 16,384 (=4^7^) possible 7-mers. Therefore, the consensus sequence of MazF-hw is rare throughout the genome. Only 183 out of 2610 open reading frames (ORFs) contain this sequence [28]. Since the list of genes carrying MazF-hw sites includes rhodopsin and genes involved in energy metabolism, MazF-hw is postulated to play a role in metabolic adaptation [28].

### 2.6. ChpBK

ChpBK from *E. coli* is highly homologous to MazF-ec, sharing a 35% sequence identity with MazF [22]. It cleaves either before or after the A-residue on ACY (Y denotes G, A or U). In a similar manner as MazF, ChpBK digestion produces 2′,3′-cyclic phosphate at the 3′ end of the 5′ product and the 5′-OH at the 5′ end of the 3′ product, demonstrating that ChpBK cleaves phosphodiester bonds of RNA [22]. Despite a high sequence similarity with MazF, ChpBK is not a potent toxin. While in vivo expression of *MazF*-ec inhibits protein synthesis almost completely, ChpBK reduces the level of protein synthesis to only 60% of normal levels [3,22]. The slower rate of mRNA cleavage is proposed to be due to its acidic pI (5.2), which leads to a weak affinity to RNA.

### 2.7. Kid (PemK)

PemK and Kid were originally thought to be distinctive toxins involved in the maintenance of separate low copy plasmids, R100 and R1, respectively [43,44,63,64,65,66]. Later, the two toxins were identified to be the same protein. The sequence-specific endoribonuclease activity, rather than direct inhibition of DNA replication initiation, is responsible for plasmid maintenance [43,44]. There is a discrepancy in the proposed consensus sequence [43,44]. The earlier study of in vitro digestion of synthetic 30-bp RNA showed that Kid digests at the 5′ and 3′ side of the A residue in UAH (H is C, A or U) at equal frequencies [43], while a later study identified the consensus to be UU^ACU [44].

### 2.8. VapC

VapC was first identified in pathogenic *Dichelobacter nodosus*, and many homologues are found in a variety of taxa, including the sexually-transmitted pathogen *Neisseria gonorrhoeae* [67,68,69]. Some bacterial genomes carry numerous copies of *vapBC* modules. For example, *M. tuberculosis* contains 45 copies of *vapC* genes [67,70,71].

VapC belongs to a different protein family from MazF. VapC consists of the PIN-domain and is similar to Mg^2+^-dependent RNases [67]. The structure of the active site is homologous to the phage T4 RNase H and to flap endonucleases, and VapC requires a single metal ion for catalysis. Toxin and antitoxin (VapB) interact physically to form the VapBC complex [67]. The VapBC complex binds inverted DNA repeats within the promoter region of the operon and regulates its own transcription [72].

VapC is a sequence-specific endoribonuclease with various RNA targets. VapC from *Shigella flexneri* 2a and *Salmonella enterica* cleave the anticodon stem loop of the initiator tRNA (tRNA^fMet^), thus inhibiting translational initiation [35]. VapC in *M. tuberculosis* cleaves the single-stranded RNA sequence with a specific secondary structure, AU^AW-hairpin-G- (W denotes U or A) [48]. The results imply that this homologue has specificity for both the sequence and secondary structure of the RNA.

### 2.9. MqsR

*mqsAR* loci are highly induced in biofilm and were originally classified as regulators of biofilm formation [73,74]. Later, it was shown that MqsR overexpression inhibits protein synthesis and that it has a ribosome independent endoribonuclease activity [49]. The *E. coli* MqsR recognition sequence is GCU and GCA [49]. Cleavage occurs at the 5′ and/or 3′ end of the G-residue. Despite exhibiting sequence-specific ribonuclease activity independently of a ribosome, MqsR belongs to the same protein family as RelE and YoeB, which are the ribosome-dependent ribonucleases [39].

MqsA, an antitoxin of MqsR, also belongs to a distinctive protein family from MazE [39,75]. There are many differences between MqsA and MazE, with regard to the structure and the mechanism of inhibiting the toxin’s activity. MqsA consists of a novel fold containing a zinc ion, although it merely serves structural role and not a catalytic role [39]. Unlike MazE, whose unstructured C-terminal tail is crucial for binding with the toxin to inhibit its activity, the structure of MqsA is not flexible [39,76]. Furthermore, MqsA does not directly bind to the active site of the toxin to block catalysis. Binding with MqsA induces a substantial conformational change in MqsR so that it loses its activity. Additionally, when bound to its antitoxin, the active sites in two MqsR proteins face one another, making them inaccessible to the target mRNA.

Despite its distinct phylogenetic origin and mechanism of toxin sequestration, MqsRA autoregulates its own transcription in a similar manner as other Type II TA systems [49,76,77]. The antitoxin or TA complex binds with DNA at the palindromic sequence and suppresses its own expression. MqsRA also regulates the expression of other genes as a global transcriptional regulator, which will be further discussed in the later sections.

### 2.10. YhaV

*E. coli* YhaV is a ribosome-independent endoribonuclease that digests mRNA, 16S rRNA and 23S rRNA in vitro in the absence of the ribosome [78]. However, its recognition sequence remains unknown. YhaV is a toxin that also belongs to the RelE superfamily, containing highly-conserved arginine residues [78]. The replacement of two of these residues results in the loss of enzymatic activity [78]. The antitoxin, PrlF, on the other hand, belongs to the same family as MazE and PemL (AbrB super family), suggesting the ancient genetic rearrangement between TA pairs and a swapping of cognate pairs [78,79,80].

### 2.11. HicA

HicA from *E. coli* digests mRNA, as well as tmRNA, independent of translation [2]. The induction of HicA reduces the rate of translation by up to 50% within 10 min [2]. Studies with primer extension did not yield a consensus cleavage sequence, but the cleavage of tmRNA at two A^AAC sites was observed [2]. Further research is needed to decipher the exact target sequence specificity and to determine whether a requirement for a specific secondary structure exists. The antitoxin HicB is degraded by Lon under amino acid starvation [2]. The *hicB* deletion mutant of *E. coli* is viable, indicating either that HicA is not a potent toxin and/or an additional protein in the genome can neutralize HicA.

### 2.12. RnlA

*E. coli* RnlA is a toxin of the Type II TA system, *rnlAB*, and it is an antagonist of the T4 phage [81]. RnlA is composed of three domains and shares no homology with known TA toxins [82]. Purified RnlA digests phage-derived mRNA in vitro [83]. However, the observed activity is low, unless a cellular fraction containing ribosomal proteins and ribosome-associated proteins are added, suggesting the possible importance of the interaction with a ribosome under physiological conditions. Many proteins are known to bind RnlA. The activity of RnlA is directly activated by RNase HI and inhibited by phage protein Dmd [84,85].

### 2.13. ToxN_Pa_, ToxN_Bt_, AbiQ

ToxN and AbiQ are toxins of Type III TA systems, while the above-mentioned ribonucleases belong to Type II TA systems. In Type III TA systems, RNA functions as an antitoxin that blocks protein toxins by forming antitoxin RNA-toxin protein complexes rather than the protein-protein complexes formed in Type II TA systems [47,86,87,88,89].

ToxN from *Pectobacterium atrosepticum* (ToxN_Pa_) cleaves AA^AK (K represents U or G), and *Bacillus thuringiensis* cleaves at A^AAAA [47,87]. Cleavage of the antitoxin RNA by AbiQ from *Lactococcus lactis* demonstrated that this enzyme also has ribonuclease activity, although the sequence specificity has not yet been determined [90]. ToxN expression inhibits cell growth, and the co-expression of the antitoxin (*toxI*) neutralizes the toxicity. Inhibition of ToxN activity by antitoxin RNA has also been confirmed in vitro [47].

The antitoxin, *toxI* of *toxIN*, is a non-coding RNA that is an oligomer of 5.5 repeats of nearly identical 36-base sequences [47]. The antitoxin RNA is digested to the monomeric fragments by its cognate toxin. The co-crystal structure of the TA complex indicates that toxin-processed antitoxin RNA remains bound to the toxin at its active site and inhibits the toxin’s activity. The toxin has a stronger affinity to unprocessed RNA than to processed RNA, suggesting the involvement of toxins in the antitoxin processing and their importance in the inhibition of toxin [47].

## 3. Physiological Roles

While the initial TA system was discovered for its role in plasmid maintenance, a majority of sequence-specific ribonucleases are encoded from chromosomes and are involved in other cellular processes [1,44,91,92,93]. The identification of the physiological roles of each TA module has been difficult due to a lack of obvious phenotypes in many single deletion strains [94]. Hypothetical functions of TA nucleases include abortive phage infection, stress response, persister formation, biofilm, pathogenicity and regulation of metabolism and translation [1,44,48,91,92,93].

How do endoribonucleases affect such diverse aspects of bacterial physiology? A common mechanism of action is through the reversible inhibition of translation, either by modulating the production of a specific set of proteins and/or by reducing the overall rate of translation. A global decrease in translation seems to play a significant role in the formation of transient cellular dormancy. The functions of antitoxins and TA complexes as global transcriptional regulators also seem to be a crucial aspect of the role of TA systems in modulating cellular physiology [95,96].

### 3.1. Plasmid Maintenance and Copy Number

The first TA system was discovered as an “addiction module” to kill plasmid-free cells [91,97]. While cells carrying TA modules continue to transcribe both TA genes to ensure sequestration of toxins from cellular targets by antitoxins, daughter cells devoid of plasmids cannot replenish the pool of antitoxins, which are preferentially degraded by proteases. The cells without plasmids will thus be removed from the population by the actions of free toxins.

The *phd-doc* module of plasmid-prophage P1 is one of the most studied modules responsible for postsegregational killing [98]. Although the exact mechanism of how the toxin Doc induces cell death of the plasmid-free cell remains unknown, inhibition of protein synthesis in plasmid-less cells has been observed [93]. Moreover, postsegregational killing by the *phd-doc* module of plasmid prophage P1 requires the presence of chromosomal *mazEF*, suggesting that the inhibition of translation by MazF and possibly other nucleases is essential for killing plasmid-free cells [93].

The direct involvement of TA ribonuclease in postsegregational killing is observed with Kid, a MazF homologue, from plasmid R1 [44]. When the copy number of R1 plasmids becomes low, Kid cleaves both host and plasmid-derived mRNAs at UUACU. One of the transcripts digested by Kid is CopA, antisense RNA that impedes the translation of the R1 replication protein, RepA. Kid activation allows for the increase in RepA, allowing for the restoration of the plasmid copy number. Simultaneously, the digestion of cellular mRNA inhibits bacterial growth until the plasmid copy number has increased.

### 3.2. Regulation of Translation

The sequence-specific cleavage by an endoribonuclease would allow differential digestion of unique sets of gene transcripts. In particular, a ribonuclease with a relatively long recognition sequence (e.g., seven-base cutter MazF-hw) would only cleave a minor fraction of the cellular mRNA. For example, the MazF-hw recognition sequence is found only within 7% of coding sequences [49]. Additionally, the RNA-seq of all cellular mRNA has shown that not all of the recognition sequences are digested by corresponding nucleases in vivo [32]. The limited number of actual in vivo cleavages could possibly allow regulation of even fewer sets of genes and/or different sets of genes that share a physiological function.

The digestion of rRNA, tRNA and tmRNA and/or cleavage of the majority of mRNA has an impact on the global rate of translation. Slowed translation can have multiple consequences. When nutrients are limited, the inhibition of translation would dampen the energetic cost by lowering unwanted anabolic processes. It would also allow for the maintenance of metabolic balance when other processes are inhibited [99]. Simultaneous digestion of mRNA at or near the SD sequence and aSD portions of 16S rRNA may also allow for the preferential translation of specific sets of genes by translating the resulting lmRNA by the stress ribosomes [32].

### 3.3. Auxiliary Transcriptional Regulation

The mode of action of TA systems is not limited to the nuclease activities of the toxins. As a global transcriptional regulator, some antitoxin and/or TA complexes can alter the gene expression patterns of many genes. MqsRA was shown to bind to the palindromic sequence within the promoter regions of non-TA genes, including *rpoS* and *cspD*, therefore having an influence on processes such as stress response, motility and biofilm formation [39,41]. Deletion of *mqsR* resulted in the repression of 239 genes and the induction of 76 genes in the log-phase, suggesting that MqsR has a genome-wide influence in gene expression either through its nuclease activity and/or via MqsA [41]. The antitoxin DinJ of YafQ (ribosome-dependent nuclease) also regulates non-TA genes as a transcriptional regulator [42]. DinJ binds to the LexA palindrome within the promoter of *cspE,* a cold shock protein [42]. As CspE plays a role in the translation of *rpoS* mRNA, DinJ influences *rpoS* expression through *cspE* [42].

The toxin’s role in transcriptional regulation is to influence the stability of the antitoxin and its binding capacity to DNA [40]. When RelBE proteins are produced at the proper stoichiometric ratio, two TA complexes cooperatively bind DNA to repress the expression. The presence of excess toxins (RelE) abolishes cooperative binding, causing de-repression of transcription to occur [40]. Preferential proteolysis of antitoxins by stress-inducible proteases must alter the toxin-antitoxin ratio, leading to a change in efficacy of transcriptional regulation by these complexes.

### 3.4. Phage Abortive Infection

Phage-abortive infection (Abi) is a type of phage resistance that induces the death of a single infected cell to ensure the survival of the remaining population [100]. This altruistic behavior of a single cell involves cell growth arrest after phage infection, caused by several different TA systems. Each TA protects against specific types of bacteriophages. The first TA loci that were identified to give resistance to phages comprised the Hok/Sok system on *E. coli* plasmid R1 [101]. Pore-forming toxin Hok inhibits the propagation of T4 phage through the induction of membrane depolarization.

Many sequence-specific ribonucleases have also been shown to provide protection against phage propagation. The activity of the TA toxin RnlA is induced after T4 phage infection. RnlA cleaves most of the cellular and phage-derived mRNA in the absence of the antitoxin RnlB, hence blocking the reproduction and spreading of T4 phage [85,102,103,104,105]. *mazEF* antagonizes the induction of phage T4, as well as P1 [106,107]. *toxIN* protects cells from multiple phages [88]. The digestion of mRNA is also the likely molecular basis behind *mazEF* and *toxIN*-led abortive infections.

Since phages have genes on their genomes that specifically antagonize TA systems, it can be deduced that TA modules do indeed protect the hosts from phage propagation. Dmd protein, encoded from T4 phage, allows phage propagation in host cells by suppressing RnlA [83,85]. Upon deletion of *dmd*, phages become sensitive to the RnlA activity and cannot propagate. Direct inhibition of RnlA activity by Dmd is shown both in vivo and in vitro [83,85,108]. T4 phages also encode ADP-ribosyltransferase (Alt) that can modify MazF [107]. ADP-ribosylated MazF has shown a reduced catalytic activity, thereby unable to block phage propagation [107]. Phages that are capable of escaping *toxIN*-mediated abortive infections carry sequences similar to the antitoxin sequence. The study of mutants of phage TE that are insensitive to the *toxIN* system of *P. atrosepticum* 1043 identified the expansion of repeats similar to repeats in antitoxin *toxI* [109]. By expressing antitoxin-like RNA sequences, phage TE mutants can escape the Abi by blocking the ToxN toxicity [109]. Phage T7 neutralizes TA-based defense systems by producing a protein, Gp4.5, which directly interacts with Lon and inhibits its activity [110].

### 3.5. Cell Death Pathways (DNA Replication Related)

Starvation for thymine induces a specific type of cell death, called thymine-less death (TLD) [111]. While TLD was discovered over 60 years ago, the exact molecular mechanism behind TLD remains unknown. The lack of thymine in media induces DNA damage and DNA structural abnormalities [99,112,113,114]. The continuation of RNA and protein synthesis, despite the cessation of DNA synthesis, also seems to be crucial for TLD, as the inhibition of RNA and protein synthesis rescues cells from undergoing TLD [99]. Fonville and colleagues suggested that the lack of thymine can induce a RecA/LexA-dependent SOS response [115]. Later, Erental and colleagues proposed that depending on the severity of DNA damage, RecA/LexA can mediate an apoptosis like death (ALD) or the SOS-response [116].

The exact role of TA systems on TLD also remains unclear. One study showed that during the growth in thymine-less media, the transcription of the *mazEF* module became diminished, which led to the activation of genes such *as yfbU*, *ygcR*, *clpP*, *slyD* and *yfiD* [117]. Conversely, Erenal et al. showed that the transcriptional level of *mazEF* increased in absence of thymine [116]. Additionally, while Nakayama and colleagues showed an inhibition of translation rescues TLD, Erenal’s model suggested that the induction of nucleases led to cell death. Further investigation is required to decipher the exact mechanisms and physiological roles of each pathway.

Replication arrest, caused by hydroxyurea (HU), induces DNA-damage independent cell death [118,119]. HU inhibits class I ribonucleoreductase, which is required for the formation of deoxyribonucleotide triphosphate (dNTPs). Depletion of dNTPs results in the inhibition of DNA replication [119]. HU also upregulates iron uptake genes, which leads to increased hydroxyl radicals through the Fenton reaction [120]. The increased formation of hydroxyl radicals is crucial for HU-induced cell death in *E. coli* [120]. The expression of TA toxins is upregulated in HU-treated cells [121]. *mazEF* is important in HU-induced cell death, as the deletion of *mazEF* increased the cells’ resistance to HU [120]. Furthermore, other TA systems, such as *relBE*, *mqsR* and *higAB*, are also postulated to be important in HU-induced cell death [120]. Davies et al. proposed that increased iron uptake and an increased membrane stress induced by MazF/RelE action are both required for hydroxyl radical-induced cell death [120].

### 3.6. Stress Response

The gene expression of many TA systems can be altered under various stress conditions [2,4,122,123]. For instance, antibiotics such as chloramphenicol, mitomycin C, kanamycin and ciprofloxacin activate the transcription of TA systems, including *mazEF* [19,122,123,124,125]. Glucose starvation, chloramphenicol, as well as amino acid starvation induce *hicA* expression [2]. Oxidative stress and ampicillin application induce *mqsRA* [126]. MqsRA is also implicated in deoxycholate tolerance [127].

In particular, the role of MqsA in general stress responses has been well documented [96]. As a DNA binding protein, MqsA binds the promoter region of *rpoS*, as well as other promoters [39,41]. RpoS is a stress-responsive alternative sigma factor, controlling approximately 500 genes in *E. coli* in response to a variety of stresses [128]. MqsA is a negative regulator of *rpoS* transcription, and deletion of *mqsA* induces the expression of *rpoS*, which results in an increased stress resistance [96]. Conversely, overexpression of MqsA, both in wildtype and in *mqsRA* deletion strains, lead to a decrease in oxidative stress, suggesting that MqsA affects stress resistance, independent of the toxin MqsR [96]. This effect is proposed to be mediated by affecting the expression of *rpoS*, as *rpoS* controls the expression of catalase, an essential enzyme for oxidative stress response [96].

Stringent response, caused by amino acid starvation, induces the expression of more than ten TA systems, including *mazEF* [2,4,122]. The exact role of TA ribonucleases in stringent responses is still under discussion. Although the digestion of cellular mRNA by induced toxins has been observed, Christensen and colleagues proposed that the majority of mRNA digestion is done by the stalled ribosomes [4]. TA toxin-mediated digestion may merely serve as an additional mechanism to further inhibit translation under extreme starvation conditions.

VapBC6 of hyperthermophilic *Sulfolobus solfataricus* is crucial for their thermophilicity [129]. When *vapBC6* is deleted, cells become heat-sensitive, and the growth rate at elevated temperatures is reduced. The expression of the toxin VapC6 is induced at high temperatures, and its activity is temperature dependent. The enzyme shows full, partial and almost zero activity at 80 °C, 65 °C and 37 °C, respectively. A preferential in vivo degradation of a few gene transcripts (*ddpB-1*, *tetR* and *vapB6*) by VapC6 has been detected, but its exact role in thermostability remains to be elucidated [129].

As a DNA binding protein, PrlF controls the expression of the *prlF-yhaV* operon [78]. The *prlF* mutant allele (*prlF1*) was isolated as a suppressor of *htrA* (*degP*) null phenotype [130]. HtrA is a periplasmic protease that is essential for cell growth at higher temperatures [131]. The *prlF1* loci also suppress the lethality caused by the clogging of SecYEG translocator with LamB-LacZ [132]. The activation of Lon is postulated to be important for these phenotypes. These results suggest the importance of *yhaV*-*prlF* under stress conditions, particularly stress that induces ruinous membranes.

The activation of HicA, YafQ and HigB suppresses the lethality caused by the loss of sigma E, an alternative sigma factor for extracytoplasmic stress response in *E. coli* [133]. The exact mechanism, however, remains unknown.

### 3.7. Persister

The importance of TA systems on the production of dormant cells has been implicated [134]. Due to the stochastic nature of the growth state variation, partially due to TA induction, patterns among the members of a community, a fraction of the population becomes transiently metabolically inactive [135,136]. These dormant cells are insusceptible to stresses, such as antibiotic treatment, and are called persisters [137]. A higher level of TA toxins including *dinJ*, *yoeB*, *yefM*, *relE*, *chpA* and *mqsR* is also observed in non-dividing persister cells [124].

A TA module, *hipAB*, was originally proposed to be linked to persister cell formation [138,139]. In this particular model, the HipA concentration above a threshold level determines the number of persister cells and the duration of dormancy in a ppGpp-dependent manner [140]. HipA phosphorylates and inactivates glutamyl tRNA synthetase [141]. The resulting accumulation of the stalled ribosome increases ppGpp through the activation of ppGpp synthetase I (RelA). Additionally, HipAB can bind to the promoter region of *relA*, possibly modulating the amount of RelA, thereby ppGpp level [142]. Lon-dependent degradation of TA antitoxins is crucial for HipA-induced persister formation, as it depends on the activation of at least ten other TA systems [136,143].

Recent studies have shown that TA toxins can increase the fraction of persisters even in the cells lacking ppGpp, albeit at lower levels [144]. Many TA toxins (MqsR, MazF, GhoT, YafQ, RelE) increase persisters when overexpressed in Δ*relA*Δ*spoT* cells, which cannot produce ppGpp [144].

It is interesting to note that the deletion of *rpoS*, as well as stress resistance genes also has been shown to increase persisters [145]. As mentioned above, MqsA modulates *rpoS* expression at the transcriptional level [96]. A number of persisters within a population can also be influenced by TisB, a TA toxin that lacks ribonuclease activity, but affects the proton motive force, [125]. Many different treatments that are associated with reduced growth rate appear to increase in persisters [145]. More study is required to decipher the genetic and biochemical basis for the formation of persisters, TA systems and the general stress response.

### 3.8. Biofilm

The *mqsRA* was first identified for its role in regulating biofilm [73]. The expression of *mqsRA* is induced by the quorum-sensing autoinducer (AI-2) [73]. MqsRA regulates the expression of many genes, including genes involved in curli and cellulose production, motility and stress resistance through the transcriptional regulation of global regulators such as *rpoS* and *cspD* [39,41]. The cold shock protein CspD is a master regulator of curli and cellulose production, which are the main constituents of biofilm matrices [146,147,148]. MqsA binds palindromic sequences in the promoter region of *cspD* and represses its expression under favorable growth conditions [41]. Under stress, such as nutrient limitation, MqsA is degraded by proteases. As a result, the *cspD* expression will be de-repressed, leading to the production of curli and cellulose [39,41]. Simultaneously, the degradation of MqsA also leads to the induction of sigma S expression, which in turn represses the expression of the motility gene via FlhDC, which also favors cells to form biofilms [73].

Other TA systems, whose toxins do not possess nuclease activities, are also implicated in biofilm formation. In *B. subtilis*, TpxA and YqcG toxins are expressed while under nitrogen limiting conditions and are important for the proper development of biofilms [149]. YafQ affects the number of cells in biofilm that survive antibiotic treatment [150]. These results demonstrate plausible links between biofilms, stress response and TA systems.

### 3.9. Virulence/Symbiosis and Metabolism

Many pathogens, as well as symbiotic bacteria carry multiple types of TA systems that are implicated in host cell entry and in adaptation to intracellular environments. FitB toxin of FitAB from *Neisseria gonorrhoeae*, a structural homologue of VapC, is crucial for pathogens to efficiently cross the epithelial barrier to enter host cells [151]. The *fitAB* deletion mutant exhibits much slower rates of crossing polarized monolayer membranes than the wildtype stain [151].

Genome sequence comparison of *M. tuberculosis* and its non-pathogenic relative, regarding MazF-mt3 and MazF-mt7 cleavage sites, revealed a potential connection between these toxins and the pathogenicity of *M. tuberculosis* [21,45]. Several proline-proline-glutamic acid (PPE) genes in the *M. tuberculosis* genome show an underrepresented frequency of MazF-mt3 and MazF-mt7 cleavage sites [45]. The PPE family consists of proteins with unknown functions that localize on cell walls or cell surfaces [152,153]. While non-pathogenic *Mycobacterium* strains lack PPE genes, pathogenic *M. tuberculosis* has multiple copies of PPE genes. This evidence suggest their involvement in host-pathogen interactions and the pathogenicity of *M. tuberculosis*. A biological basis behind the lack of MazF-mt3 and MazF-mt7 cleavage sites in PPE genes and their roles in immunopathogenicity await experimental verification.

In *Mycobacteria*, VapBC plays a role in coupling cell growth and carbon metabolism [48]. When *vapBC* is deleted, cells consume glycerol at a faster rate than the wildtype, without increasing cell growth. Additionally, the expression of genes in sugar metabolism and transport is specifically altered when VapC is overexpressed.

VapC homologue NtrR in *Sinorhizobium meliloti* is implicated in maintaining the metabolic balance and adaptation to metabolism inside of the symbiotic host [154]. The *ntrR* mutant exhibits a loss of the ability to effectively down-regulate the genes involved in symbiosis and metabolism under microoxic conditions. In particular, the transcription of *nod* (nodulation) and *nif* (nitrogenase) genes is elevated in the *ntrR* knockout mutant. As a result, the *ntrR* deletion strain shows an increase in nitrogen concentration and biomass, as compared with the wildtype strain [154,155].

## 4. Network of TA Systems

Genome-wide studies of TA systems uncovered a specific and extensive network of cross-activation and inhibition between TA systems [156,157]. Some interactions are mediated by the proteomic degradation of antitoxins by proteases (such as Lon), while others are independent of proteolysis.

### 4.1. Lon Dependent Degradation of Antitoxin

A well-studied role of stress-inducible proteases in TA systems is their ability to preferentially degrade antitoxins, which frees toxins to exert toxic functions only under stress conditions [2,15,158,159]. The degradation of antitoxins, which is a negative transcriptional repressor, also leads to the induction of the expression of its own module and other genes [57,78,136].

In addition, Lon also seems to play a role in the cross-regulation of multiple TA systems. A study of *Salmonella* and *Shigella* showed that VapC overexpression induces *yefM-yoeB* in a Lon protease-dependent manner [157]. HipA-mediated persister formation requires antitoxin degradation by Lon to activate multiple TA toxins [136]. Activation of Lon by overexpression of TA genes seems crucial for these phenomena. A variety of TA genes can activate Lon, for example ectopic expression of the antitoxin *prlF* of *prlF-yhaV* has been shown to activate Lon [78,132]. A mutation on *prlF* (*prlF1* allele) also leads to Lon activation [132]. Although the consequence of such activation on regulation of other TA genes has not been directly examined, the degradation of antitoxins and release of cognate toxins are expected.

### 4.2. Transcriptional Cross-Activation

Activation of one TA module has been shown to induce the transcription of specific sets of TA modules in a Lon-independent manner, as well. Using a *lon* deletion strain, Kasari and colleagues demonstrated that RelE expression leads to the induction of the transcription of *mqsRA*, *mazEF*, *dinJ-fayQ*, *hicAB*, *yefM-yoeB* and *prlF-yhaV* within 1 h [156]. In return, the expression of toxins MazF, MqsR, HicA and HipA leads to the induction of *relBE* operon. In contrast, induction of YafQ, a ribosome-dependent ribonuclease, did not induce *relBE* overexpression, showing a specificity in the pattern of cross-activation. These activations do not require Lon or other proteases (e.g., Clp). An analysis of transcriptomes showed that the overproduced toxins indeed digested the target TA transcripts [156]. mRNA fragments were further digested at different rates within the toxin and antitoxin-encoding regions. mRNA fragments containing antitoxin genes were preferentially digested, which lead to the accumulations of fragments containing toxin gene sequences from which toxin proteins were successfully translated [156].

These cross-links do not seem to be an artifact of ectopic expression, but have physiological relevance. Amino acid starvation activates *relBE*, which in turn activates *mazEF* [156]. In the *relBE* deletion strain, *mazEF* induction does not occur during amino acid starvation. While *mazEF* induction during the stringent response is dependent on *relBE*, the induction of *mqsR* under the same conditions does not depend on *relBE* despite *relBE* being able to induce *mqsR* when *RelBE* is over produced. Similarly, the overexpression of *doc* toxin transactivates *relBE* expression [160]. MqsR activates the expression of *ghoT* by preferentially cleaving the antitoxin-encoding portion of the *ghoS-ghoT* mRNA [126].

### 4.3. Other Regulation

The neutralization of toxin proteins by non-cognate antitoxin proteins has been observed in *M. tuberculosis* [161]. Several non-cognate pairs of toxins and antitoxins have shown physical interactions. This raises the possibility that changing the level of one TA system can influence the activity of multiple other TA proteins within a single organism. In contrast, another study claimed no interaction between non-cognate pairs of VapBC from a single organism, despite an evolutionary similarity between the tested TA pairs [71]. Whether or not these interactions have physiological relevance needs to be further examined.

## 5. Remarks

The complex physiological changes listed above are often controlled by one or more TA toxins. Multiple TA modules are activated during one response, and cross-regulation among TA modules adds layers of complexity. The lack of obvious phenotypes in single TA deletion strains has placed hurdles for determining the physiological role of each TA system. Some of the contradicting data obtained may be due to the use of ectopic expression, which inevitably leads to the activation of Lon and/or cross-activation of other TA systems.

In order to achieve a full understanding of the complex physiological phenomena controlled by sequence-specific endoribonucleases, further research on each toxin as well as on groups of TA systems is required. We are far from understanding both the extent and types of RNA degradation (e.g., how much of mRNA vs. tRNA is digested in vivo) and the importance of cross-regulations. The reversibility of certain digestions by ligases also needs to be considered when analyzing the dynamics of translational control by TA systems.

## Figures and Tables

**Table 1:** Sequence-specific ribonuclease toxins.

Toxins	Types	Sources	Consensus Sequences (Reference)	Substrates			
				mRNA	rRNA	tRNA	tmRNA
MazF/ChpBK							
MazF-ec	II	*E. coli*	A^CA [3]	+	16S		
ChpBK		*E. coli*	^ACY and A^CY [22]	+			
Kid (PemK)		R100 and R1 plasmid	U^AH and UA^H [43]	+			
			UU^ACU [44]				
MazF-mt1		*M. tuberculosis*	U^AC [21] *^1^	+			
MazF-mt3		*M. tuberculosis*	U^CCUU [26]	+	16S, 23S		
MazF-mt6		*M. tuberculosis*	UU^CCU [24]	+	23S		
MazF-mt7		*M. tuberculosis*	U^CGCU [45] *^2^	+			
MazF-mt9		*M. tuberculosis*	UU^U [25]	+		+	
MazF-mx		*M. xanthus*	GU^UGC [46] *^3^	+			
MazF-hw		*H. walsbyi*	UU^ACUCA [28]	+			
MazF-bs		*B. subtilis*	U^ACAU [27]	+			
MoxT		*B. anthracis*	U^ACAU [31]	+			
MazF		*S. aureus*	UACAU [29]	+			
MazF		*S. equorum*	U^ACAU [30]	+			
ToxN_Pa_	III	*P. atrosepticum*	AA^AK [47]	+			
ToxN_Bt_		*B. thuringiensis*	A^AAAA [47]	+			
AbiQ		*L. lactis*	n.d.	+			
Homologue of phage t4 RNase H with PIN domain							
VapC	II	*M. tuberculosis*	AU^AW-hairpin-G- [48]	+			
VapC		*S. flexneri* 2a and *S. enterica*	Anticodon stem-loop of tRNA^fMet^ [35]	-	-	+	-
RelE super family							
MqsR	II	*E. coli*	GCU and GCA [49] *^4^	+			
YhaV		*E. coli*	n.d.	+	16S, 23S		
No known homologues							
HicA	II	*E. coli*	n.d. for mRNA;	+			+
			A^AAC in tmRNA [2]				
RnlA		*E. coli*	n.d.	+			

*^1^ Single base in UA can be changed to another base; *^2^ with 1 or 2 base alterations; *^3^ additional cleavage sites with higher concentrations of enzymes; *^4^ cleavage occurs at 5′ and/or 3′ side of the G-residue.
